# Common cold

**DOI:** 10.3389/falgy.2023.1224988

**Published:** 2023-06-22

**Authors:** Ronald Eccles

**Affiliations:** Cardiff School of Biosciences, Cardiff University, Cardiff, United Kingdom

**Keywords:** common cold, respiratory viruses, chilling, crowding, vitamin C, vitamin D

## Abstract

The common cold is a unique human disease, as it is arguably the most common disease and because of the large number of respiratory viruses causing colds it is one of the most complex of human diseases. This review discusses the respiratory viruses and notes that all these viruses may cause the illness complex recognised as the common cold. The common cold is discussed as part of the “iceberg concept” of disease which ranges from asymptomatic infection to severe illness and death. The factors influencing the incidence of colds are discussed: crowding and sociability, stress, smoking and alcohol, immune status, sex, age, sleep, season, chilling, nutrition and exercise. The mechanism of symptoms related to the innate immune response is explained and symptomatic treatments are tabulated. Morbidity associated with common cold is discussed and possible vaccines.

## Introduction

**Figure 1:**
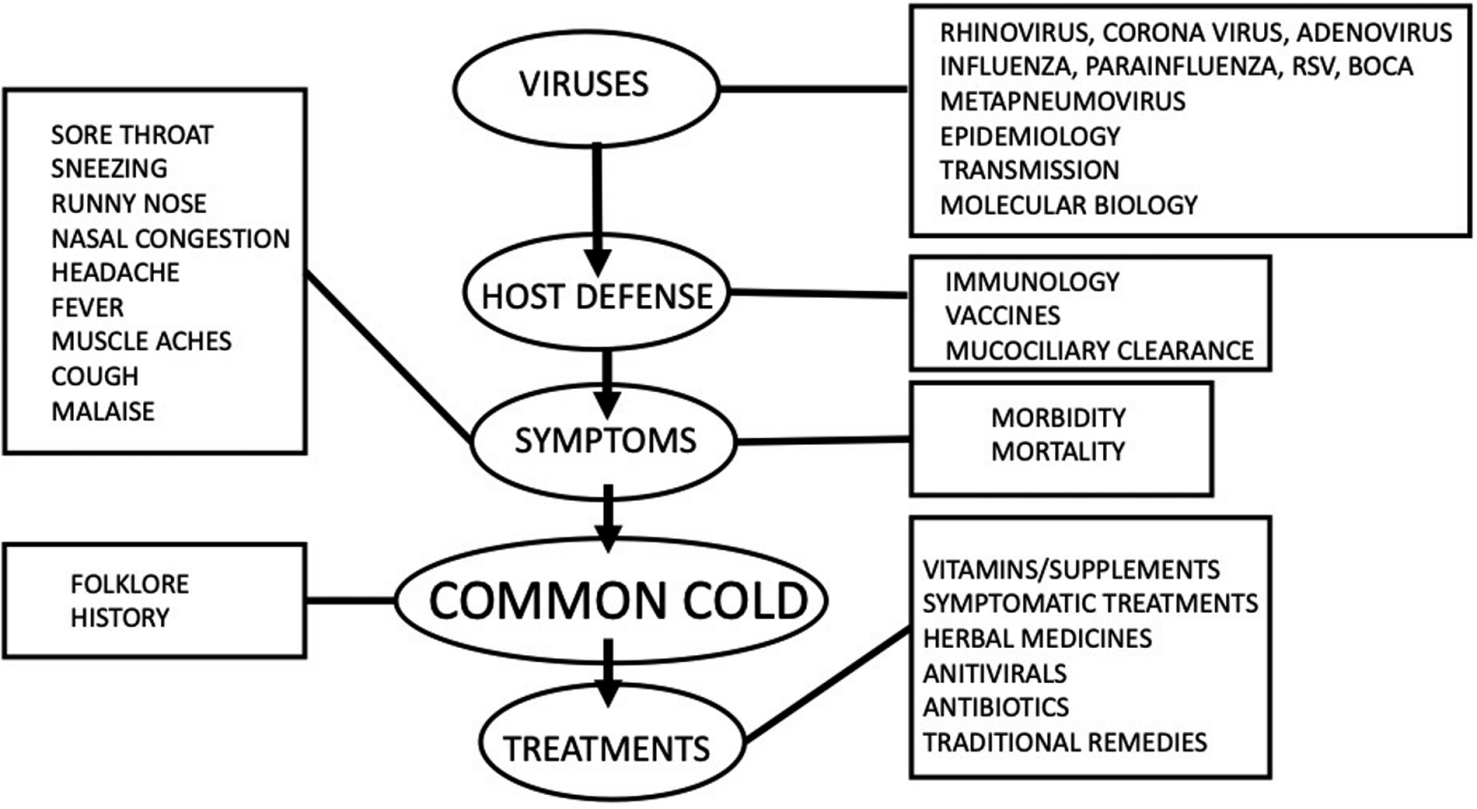
Various topics that could be discussed in a review of the “Common cold”.

A review on the common cold may look at one or more of a wide range of topics as illustrated in Figure 1 from the viruses which cause common cold to folklore and traditional remedies for common cold. The author of this review spent 30 years as the Director of the Common Cold Centre at Cardiff University UK and his expertise is mainly related to the mechanism of symptoms of common cold and treatments for common cold. This review will therefore focus on symptomatology and treatments but will also discuss new developments in this field.

The common cold is a unique human disease, as it is arguably the most common disease and because of the large number of respiratory viruses causing colds it is one of the most complex of human diseases. Because the common cold is a syndrome of symptoms and not a specific disease, even the definition of what constitutes a common cold is debatable as discussed below.

## Definition of common cold

**Figure 2:**
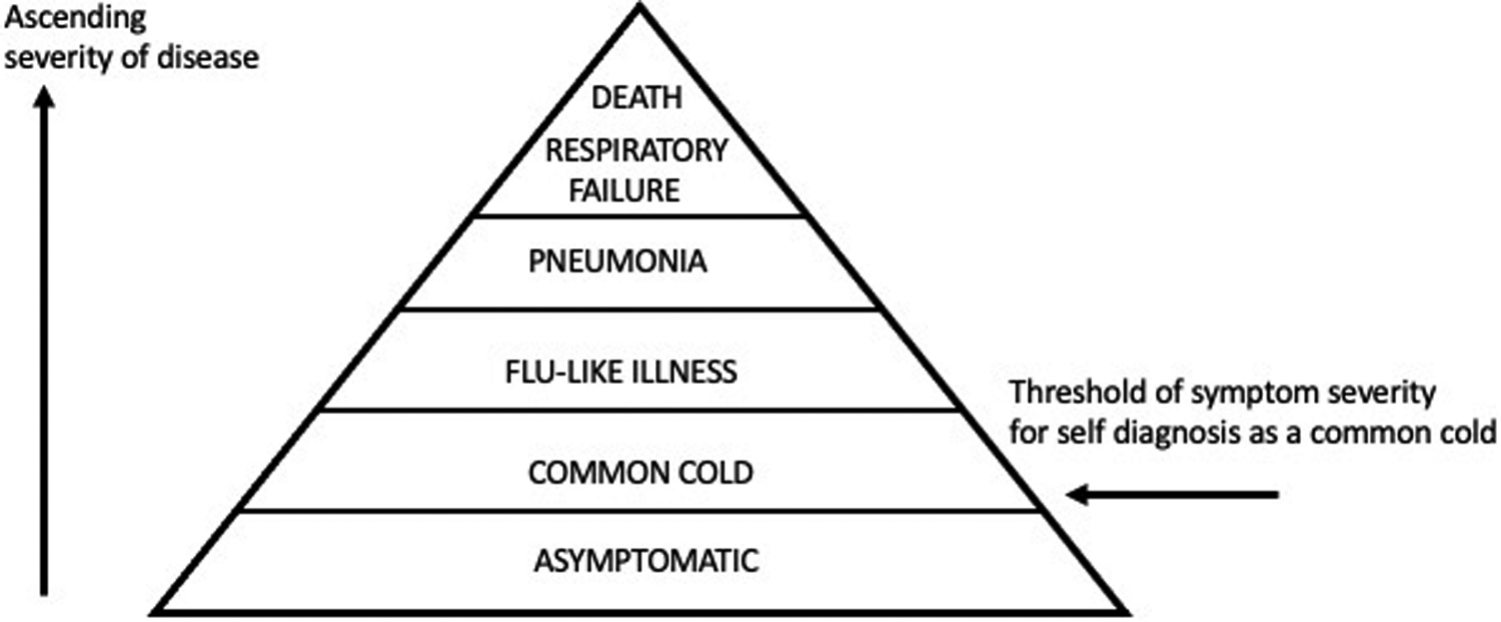
Iceberg concept of viral infection. Most infections result in asymptomatic or mild symptoms which may not reach a threshold to be self-diagnosed as a common cold. These infections are hidden and hence “below the water line of an iceberg”. Severe symptoms may reach a threshold to be termed “flu- like illness”and lower respiratory tract infections such as bronchiolitis and pneumonia may not be appreciated as often starting as a common cold.

A common cold is defined by the National Institute for Health and Care Excellence (NICE) as “a mild, self-limiting, upper respiratory tract infection characterized by nasal stuffiness and discharge, sneezing, sore throat, and cough” (1). The term “common cold” is widely used in the medical literature, but it has been argued that the common cold is more of a cultural concept rather than a clinical entity as the disease is usually self-diagnosed and treated by the patient (2). The term common cold has been used for centuries, and it is often associated with a belief that exposure to cold causes the symptoms of disease (3). It is only since the discovery in the 1950's that respiratory viruses such as rhinoviruses (4) cause the common cold that there has been a scientific explanation for this disease. “All known respiratory viruses are able to produce the illness complex recognised as the common cold” (5). But the illness complex or syndrome of symptoms that is recognised by the patient as a common cold is only part of what is often called the iceberg of virus infection as illustrated in Figure 2. Most respiratory virus infections result in a sub-clinical or asymptomatic infection which is not noticed by the host as the virus is cleared in a few days by the immune system. The iceberg concept of infection is illustrated by a study on 214 healthy individuals (age 3–63 years) (6). Nasopharyngeal swabs and symptom scores were taken twice a week for two years. Of 4,215 samples collected, 737 were positive for one or multiple respiratory viruses (influenza viruses, respiratory syncytial viruses, human rhinoviruses, coronaviruses, adenoviruses, parainfluenza viruses, human metapneumovirus) and amongst the positive results, 69%–74% of the samples were classified as asymptomatic. The iceberg concept of viral infection predicts that after asymptomatic infections the next most common syndrome is an acute self-limiting illness the “common cold”, with symptoms such as sore throat, runny nose, nasal congestion, cough and sneezing as illustrated in Figure 2. The common cold is often referred to as a “head cold” meaning the symptoms are located in the upper airways, whereas more severe “flu-like illness” as illustrated in Figure 1 involves systemic symptoms such as fever, muscle aches and pains and malaise. On infection with a respiratory virus, factors such as old age, low immune deficiency and poor nutrition may allow more severe respiratory illness such as bronchiolitis or pneumonia and less commonly result in death represented by the tip of the iceberg. Infections occur frequently and they are usually milder after several infections. Each infection does not lead to the induction of protective immunity. The common cold can therefore be considered as part of a spectrum of disease caused by respiratory viruses as illustrated in Figure 2.

## Common cold viruses

As stated above, all respiratory viruses may cause symptoms recognised by the patient as a common cold (5), and viruses that are not classified as respiratory viruses may also cause common cold symptoms when they infect the upper airways such as measles viruses (7) and enteroviruses (8), and some bacterial infections may also cause common cold symptoms (9). Table 1 lists the different groups of respiratory viruses. Rhinoviruses are the most common cause of common cold as they are found in more than half of upper respiratory tract infections and can be considered as the most common infection of humans world-wide (8). Table 1 lists the respiratory viruses known at present, but it is likely that new viruses await discovery. The metapneumovirus was discovered in 2001 by researchers in the Netherlands who identified the virus in stored nasopharyngeal samples from children, and it was later determined that his virus had been in circulation for some 65 years and that nearly every child would have been infected by metapneumovirus by the age of five (10). Human bocavirus was discovered in 2005 from nasopharyngeal samples in children and has now been shown to cause common cold in children (11).

**Table 1:** Respiratory viruses.

Virus	Abbreviation	Nucleic acid	Classification	Structure
Rhinovirus	HRV	RNA	Species A, B, C with 100 serotypes	Non-enveloped icosahedral capsid
Coronavirus Seasonal	HCoV	RNA	Types OC43, 229E, NL63, HKU1	Enveloped
Coronavirus Pandemic	SARS, MERS SARS-CoV2	RNA	Variants such as Omicron	Enveloped
Respiratory syncytial virus	HRSV	RNA	Groups A and B	Enveloped
Parainfluenza virus	HPIV	RNA	Types 1, 2, 3, 4	Enveloped
Influenza virus	Flu	RNA	Types A, B, C, D	Enveloped
Adenovirus	ADV	DNA	51 serotypes	Non-enveloped icosahedral capsid
Metapneumovirus	HMPV	RNA	Groups A and B	Enveloped
Bocavirus	HBoV	DNA	2 lineages	Non-enveloped icosahedral capsid

## Risk factors

The common cold is the most common human disease and the factors influencing susceptibility to developing common cold symptoms have been discussed for thousands of years, and form a very large literature of science and folk-lore (12). The Greeks and Romans explained common cold in terms of the so-called humoral pathology explained by Hippocrates with chilling of the body causing a disturbance of the humors and flow of mucus from the nose (12). The role of chilling as a predisposing factor to common cold persisted through to the seventeenth century and Richard Lower an eminent physician in London, UK published a treatise entitled De Catarrhalis (3) and concluded “Finally if one wishes to prevent this evil altogether nothing is more useful as a precaution than to be well protected against external cold and make sure of proper perspiration, even (should the situation demand it) by moving to a hot and dry climate. On these measures depend largely the preservation of our health and the prevention of catarrhs.” Chilling of the body surface as a cause of common cold symptoms or as a means of increasing susceptibility to infection persisted throughout the nineteenth and twentieth centuries (13) and even in the present is still proposed as a mechanism that may influence susceptibility to infection and common cold although with poor evidence and scientific explanation (14).

**Figure 3:**
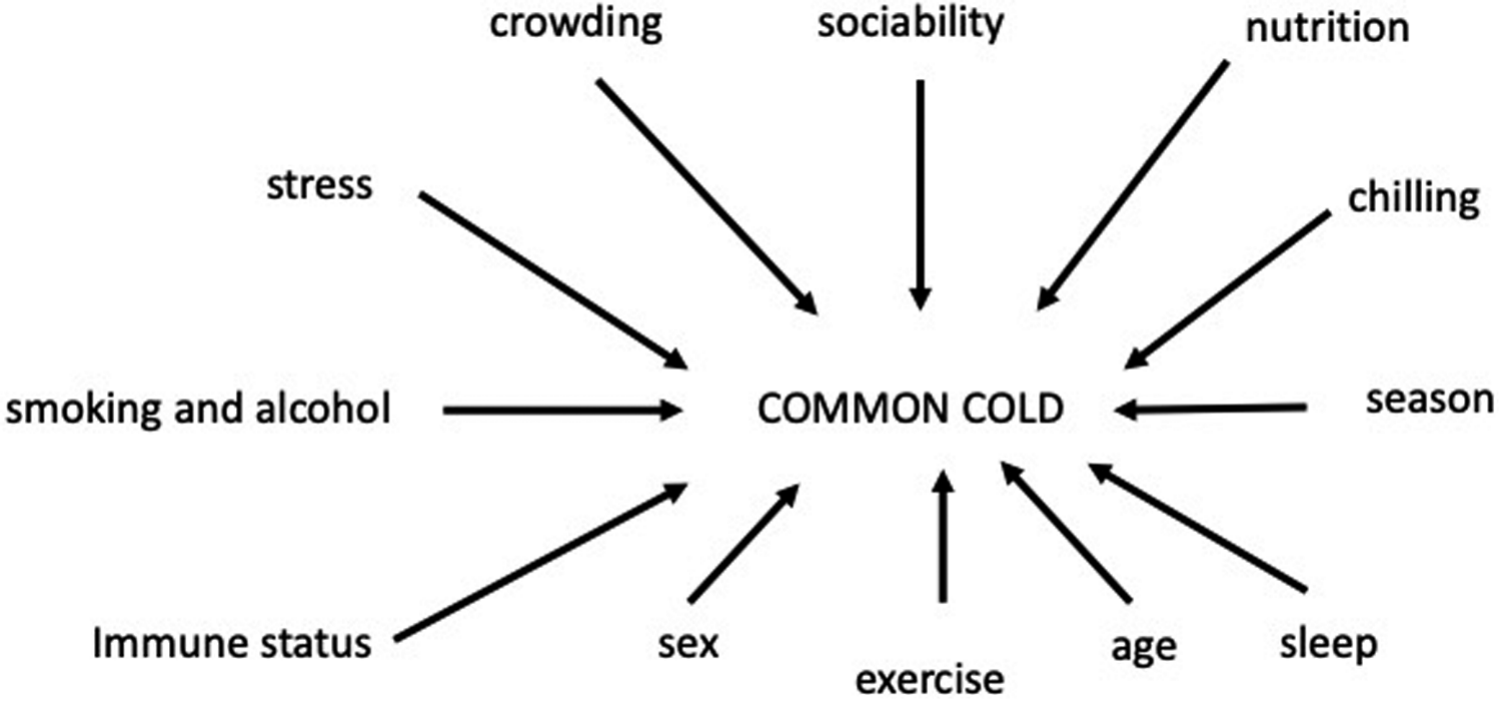
Factors that may influence susceptibility and incidence of common cold.

A discussion of the main factors that influence the incidence and susceptibility to common cold are summarised in the present review as illustrated in Figure 3 and will be discussed below.

### Crowding, sociability

Humans are infected by respiratory viruses that originate from the respiratory tract of other humans and are transmitted in respiratory mucus by aerosols generated by coughing and sneezing and by fomites such as commonly touched surfaces (15). It is self-evident that transmission of viruses will be more likely in crowds of human beings when they are close together such as in schools, colleges, cinemas, theatres, public transport, etc. (16) and social distancing and closure of crowded places and events has been used as a means of controlling spread of SARS-CoV-2 virus during the recent pandemic. Using data collected from 626 participants in England and Wales UK it was shown that spending time in underground trains, supermarkets, theatres, cinemas, concerts, restaurants or attending parties increased the risk of acquiring circulating acute respiratory infections (16). It may be assumed that sociable persons who have an active social life and have many contacts with other persons have an increased risk of common cold but studies report that the opposite may be the case with sociable persons having greater resistance to respiratory infection (17). In a study on 334 participants in Pittsburgh USA a questionnaire was used to determine measures of sociability and then the participants were challenged with a rhinovirus. Increased sociability was associated in a linear fashion with decreased probability of developing a cold and this association was independent of baseline immunity to the virus (17). Sociability was associated with better sleep and diet and more positive and less negative emotions, but analysis failed to support any of these as potential mediators and the mechanism of how sociability influences susceptibility to infection is still unknown.

### Stress

Stress is understood generally but there are disagreements about a scientific or clinical definition of stress. Stress has been defined as when external demands (stressors) exceed the capacity of the organism to adapt to conditions and cause detrimental psychological and biological changes (18). Long-term psychological stress has a negative impact on the immune system and mucosal immunity and supresses the production of secretory immunoglobulin A (sIgA) (19). Psychological stress has been shown to be a risk factor for common cold (18, 20). In a one year study carried out in a Spanish university the relationship between stressful life events and occurrence of common cold was investigated among 1,149 subjects aged between 23 and 68 years with 46% females, and 365 cases of common cold (18). The study found a moderate correlation between stress and incidence of common and concluded that the findings suggest that psychological stress is a risk factor for common cold (18). Studies on the relationship between psychological stress and common cold are difficult because stress can also be associated with changes in diet, behaviour, smoking, alcohol ingestion etc. which may also influence the risk for common cold.

There is some evidence that release of cortisol hormones associated with stress may compromise the immune response and make subjects more susceptible to developing common cold symptoms after infection (21).

### Smoking and alcohol

Smoking increases the risk of respiratory infection by damage and irritation of the respiratory epithelium and alteration of the structural, functional and immunologic host defences (22). Ingestion of alcohol in moderation is reported to decrease susceptibility to developing a cold by having a beneficial effect on the immune system (23–25). The effects of smoking and alcohol consumption on the incidence of common cold were studied by exposing 391 volunteers to respiratory viruses (24). In this study it was reported that smokers were more likely to develop infections after exposure to virus and also to develop illness compared to non-smokers and that taking up to three or four alcoholic drinks a day was associated with decreased risk of developing a cold in non-smokers (24).

### Immune status

The immune status of a subject can be considered as the presence of antibodies (immunoglobulins) against a specific respirator virus. When an infant is born it does not have a mature immune system and depends on immunity derived from the mother via immunoglobulins transferred to the infant via the placenta and breast milk (26). This maternal protection declines in the first year as the infant ceases breast feeding as immunoglobulins have a relatively short half-life. The immunity transferred to the infant via breast milk is dependent on exposure of the mother to respiratory viruses and during the COVID-19 pandemic circulation of common respiratory viruses was greatly decreased due to lockdown and isolation of the general population and this resulted in lower antibody levels in maternal milk for respiratory viruses such as RSV, influenza and coronaviruses (27). Antibody immunity against respiratory viruses such as RSV is short lived, and this explains the waning maternal and infant immunity against RSV during the COVID-19 pandemic when RSV almost disappeared from circulation, and may also explain the resurgence of RSV infections in many countries once COVID-19 restrictions were lifted (28).

Parenthood has been reported to decrease the chance of developing common cold symptoms when challenged with a common cold virus and there was a clear increase in protection with having more children in the family (29). It may be assumed that parenthood increases exposure to common cold viruses and parents acquire greater immunity than non-parents but this does not appear to be the case as the resistance to developing cold symptoms was independent of pre-challenge viral-specific immunity (viral antibody titre to the challenge virus) (29).

Respiratory infections and the common cold occur throughout life, and the success of these viruses is due to several factors; the large number of serotypes of viruses such as rhinoviruses, and antigenic drift in viruses such as influenza and rhinoviruses (30, 31). Recent exposure to a respiratory virus confers short lived immunity to that virus but reinfection may occur when antibody titres decline and when the virus evolves into new variants which may have a greater capacity for infection as has been observed with the many variants of SARS-CoV-2 during the recent pandemic (32).

### Sex

Sex differences in respiratory viral pathogenesis have been reviewed by Ursin and Klein (33). In general males are more susceptible to severe outcomes than females at younger and older ages. During the reproductive years from puberty to menopause females are at greater risk than males for severe outcomes from respiratory virus infections (33). It is not surprising that males and females differ in their immune responses as they have major differences in sex hormones and sexual characteristics.

There are sex differences in the perception of common cold symptoms as even after adjustment for other variables it has been reported that men are significantly more likely to “over rate” their symptoms in comparison to women (34).

Women are almost twice as likely to develop a cold compared to men when children are sick with a common cold in the family and this because women have more contact with children than men (35).

### Age

The incidence of common cold varies throughout life as immunity to respiratory viruses is acquired by repeated exposure to infection, with infants starting life with little immunity and high incidence of colds, and adults having fewer colds than infants due to previous exposure to viruses. The incidence of common cold is inversely related to age with infants having 6–8 colds a year compared to adults having 2–4 colds a year (36).

Age also affects the severity of disease with the extremes of age, infancy and old age, having more severe outcomes from disease than the middle range of adults. This difference is related to the immature immune system in infants and the waning immunity associated with old age. RSV infections are a major cause of bronchiolitis and hospital admissions for infants, and also cause severe respiratory infections in the elderly, but may only cause mild common cold symptoms in older children and adults (37, 38).

### Sleep

“Sleep quality is thought to be an important predictor of immunity and in turn susceptibility to the common cold” (39). In a study that challenged subjects with rhinovirus it was found that there was an inverse graded association of average sleep duration prior to the challenge and susceptibility to developing common cold symptoms after virus challenge (39). Those subjects with less than seven hours sleep were almost three times more likely to develop a cold than those with eight or more hours sleep (39). This finding has been confirmed in another study which reported that shorter sleep duration prior to rhinovirus challenge was associated with increased susceptibility to the common cold (40). The impact of sleep on immune markers is not fully understood, and this is not surprising considering the complexity of the immune response to infection, but some studies have demonstrated significant changes in immune markers such as decrease in the mitogen proliferation of lymphocytes after sleep deprivation (41).

### Season

The seasons of Spring, Summer, Autumn and Winter are determined by changes of day length in the Northern and Southern hemispheres and by changes such as rainfall in the Tropics where day length does not vary. Respiratory virus infections are present throughout the year, but the incidence of infection does vary with season with more symptomatic infections present in Winter and during the rainy season (42, 43). The seasonality of respiratory infections and common cold and flu is part of folk-lore and culture and many different causes for this seasonality have been proposed. Dowell and Ho (44) nicely summarised our understanding of the seasonality of respiratory disease in this quote

“Nearly every important respiratory pathogen of human beings exhibits distinct seasonal variations, yet after hundreds of years of observing and documenting this phenomenon modern science has only superficial observations and largely untested theories about the underlying causes. Is it the cold? Dry air? Crowding together of people indoors in winter? Where do pathogens such as influenza and respiratory syncytial virus (RSV) go in the summertime? Do they migrate across the equator and return the following winter, or do they remain present at low levels in human or animal populations until environmental or host conditions are suitable for re-emergence?”

One of the main changes with season is that inspired air is colder in Winter compared to Summer and a hypothesis has been put forward that breathing cold air inhibits local defences against infection in the upper airway by slowing muco-ciliary clearance and slowing the activity of leukocytes (45). Discussion of all the different factors that may influence the seasonality of respiratory infections would be too great a task for the current review and therefore the reader is directed to some of the literature on this topic (42–48).

### Chilling

There is a widely held folklore that respiratory infections are caused by chilling or exposure to draughts and damp, that in some way penetrate the body to cause illness. Common cold is believed to occur after “going outside with wet hair”, “getting one's feet wet” and “getting caught in the rain” (49). Laboratory experiments involving challenge with common cold viruses and chilling have failed to demonstrate any effect of cold exposure on susceptibility to infection (50–52). However, these laboratory experiments have been criticised as not representing a “real life” situation when subjects may be harbouring a sub-clinical infection which can be converted into a clinical infection by chilling of the body surface (53). The idea that sub-clinical respiratory infections could be converted to clinical infections was first proposed by Mudd and Grant (54) who demonstrated that chilling of the body surface caused an intense ischaemia and cooling of the pharynx and tonsils and that this reflex vasoconstriction of the airway epithelium could decrease resistance to infection and allow a sub-clinical infection to become symptomatic. Support for this mechanism of chilling is put forward in a review by Eccles (53) and by a review of the relevant literature by Mourtzoukou and Falagas (55).

### Nutrition

It is self-evident that poor nutrition with deficiency of calories protein and vitamins etc. will have an impact on general health and the functioning of the immune system and resistance to infection. Nutrients support epithelial integrity, restoration and maintenance. Malnourished children in developing countries are particularly susceptible to morbidity and mortality associated with acute respiratory infection (56). A review of the literature highlights that discussions on nutrition and susceptibility to common cold are dominated by studies on the possible benefits of vitamins C and D in the diet.

The role of vitamin C in preventing and treating the common cold has been controversial since the dual Nobel laureate Linus Pauling published a book in 1970 claiming that vitamin C prevents and alleviates symptoms of common cold (57). The claims in the book were widely criticised as based on opinion rather than scientific evidence but the idea that vitamin C was a cure for the common cold became widely accepted by the general public. Numerous clinical trials have been conducted on the effects of vitamin C on common cold and in 2013 a meta-analysis of 29 trials concluded “The failure of vitamin C supplementation to reduce the incidence of colds in the general population indicates that routine vitamin C supplementation is not justified” and “Regular supplementation trials have shown that vitamin C reduces the duration of colds, but this was not replicated in the few therapeutic trials that have been carried out.”

Vitamin D like Vitamin C has become accepted by the general public as a preventive treatment for common cold but scientific evidence to support this treatment in healthy adults is controversial with some reports showing no benefit (58, 59) and others reporting benefit, especially in those subjects with vitamin D deficiency (60, 61).

### Exercise

Moderate exercise has been claimed to have beneficial effects on the immune system and this is associated with fewer days of sickness with common cold whereas severe exercise can have adverse effects on the immune system and increase the incidence of common cold in athletes (62). However a meta-analysis of eleven trials could not determine whether exercise is effective at altering the occurrence, severity or duration of acute respiratory infections and therefore the role of moderate exercise on common cold incidence still requires more research before it can be accepted that exercise reduces the risk of catching a cold (63).

## Mechanism of symptoms of common cold

**Figure 4:**
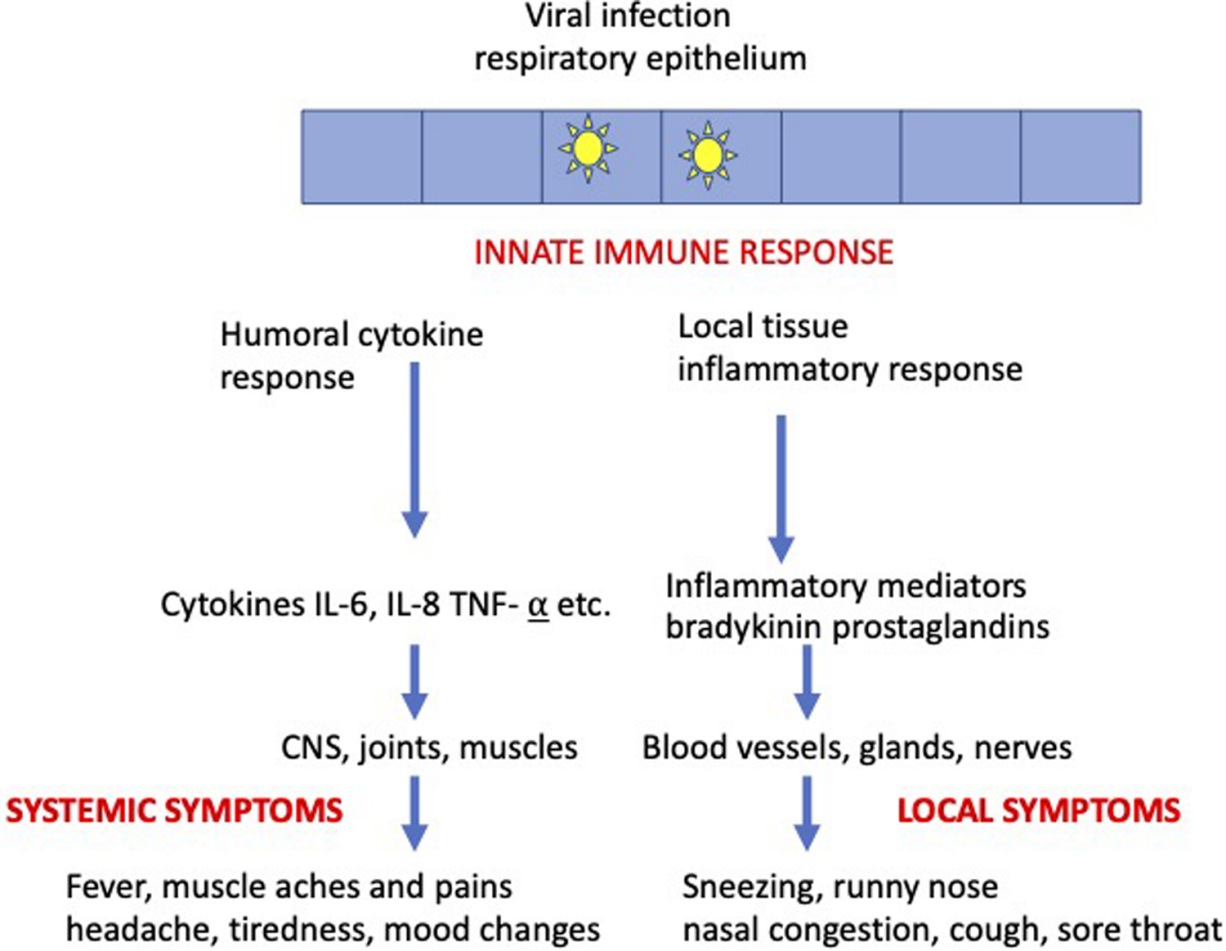
Mechanism of symptoms.

Viral infection of the upper respiratory tract triggers a local immune innate response in the infected epithelium and this response is responsible for generating all the symptoms of common cold. Prodromal symptoms at the first signs of a cold or flu include symptoms that may become more severe later in the course of the infection such as throat irritation, sneezing chilliness, fever, myalgia, headache and tiredness. The innate immune response triggers systemic and local symptoms as illustrated in Figure 4.

The systemic symptoms are mediated by cytokines such as interleukins (IL-6, IL-8, etc.) released from leukocytes and cytokines such as tumour necrosis factor alpha (TNF-α). The sensing of viral infection is by the detection of viral DNA and RNA which are detected by Pattern Recognition Receptors (PRR's) found on epithelial cells and various cells of the immune system (64). Activation of PRR's leads to the generation of cytokines such as interferons and interleukins which can circulate in the blood to cause systemic symptoms of fever, headache, muscle aches and pains, tiredness and mood changes as illustrated in Figure 4 (65).

The local symptoms are mediated by inflammatory mediators such as bradykinin and prostaglandins. Bradykinin is generated by the release of the enzyme kallikrein from epithelial cells and this enzyme converts kininogen to bradykinin (66). Bradykinin can also be generated from plasma components which act as prekallikrein (66). Bradykinin can act on blood vessels and nerves to cause symptoms of pain and sore throat, sneezing, runny nose, dilation of nasal blood vessels and nasal congestion (67, 68). Prostaglandins are generated when epithelial infection causes the release of arachidonic acid (AA) from membrane phospholipids and AA is converted to a range of prostaglandins by cyclooxygenase enzymes (69). Prostaglandin E2 is the most common of the prostaglandins and may play a major role in the generation of symptoms in viral infections (69). Prostaglandins act on blood vessels and nerves to generate symptoms of sore throat pain, nasal congestion cough and runny nose (70, 71) and they also mediate the cytokine induced symptoms of muscle aches and headache (72, 73).

## Treatments for common cold

Treatments for common cold can be considered as falling into three categories; (1) use when healthy for supporting the immune system, (2) use at first signs of symptoms with an antiviral effect, (3) use during a cold with symptom relief.

The most common treatments of the common cold are in category 3. For symptom relief and the different symptomatic treatments are listed in Table 2 and have been reviewed by Eccles (74). These medicines are marketed as over the counter (OTC) medicines that are freely available to consumers as single medicines or as multi-symptom medicines containing several actives.

**Table 2:** Symptomatic treatments for common cold.

Symptom	Treatment class	Medicines
Fever	Analgesic, antipyretic	Paracetamol (acetaminophen), ibuprofen, aspirin
Headache	Analgesic, antipyretic	As above
Muscle aches and pains	Analgesic, antipyretic	As above
Sore throat pain	Analgesic, antipyretic	As above
Sinus pain	Analgesic, antipyretic	As above
Nasal congestion	Sympathomimetics	Oxymetazoline, xylometazoline, phenylephrine, pseudoephedrine
Runny nose	Anticholinergics and sedating antihistamines	diphenhydramine, chlorpheniramine, doxylamine etc.
Cough	Antitussives and sedating antihistamines	Dextromethorphan, diphenhydramine
Sneezing	Sedating antihistamines	Diphenhydramine, chlorpheniramine, doxylamine etc.

Treatments in category (1) that support or boost the immune system consists of a wide range of vitamins, minerals, probiotics and herbal medicines that make claims, often with little scientific support, but form a very large world-wide market, which was valued at over 20 billion US dollars in 2021 and projected to be worth over 31 billion US dollars in 2028 (75). Immunity boosting and support is a large and controversial area of commerce and medicine, and specific claims are often made about the benefits in protecting against viral infections (76) which are later criticised as overstated claims (77).

A wide range of herbal medicines and plant based chemicals such as iota-carrageenan and menthol are recommended for prevention and treatment of the common cold. Echinacea and Pelargonium are two of the most popular herbal treatments and they have support for efficacy from clinical trials on common cold (78–81) although the clinical relevance of the efficacy has been reported as questionable (82). Iota-carrageenan has been studied several clinical trials on common cold and there are claims that it is an effective anti-viral medicine (83, 84).

Because common cold is such a common disease and because it is self-diagnosed and self-medicated there is a large market for cough and cold treatments worth over seventy billion US dollars in 2023 (85) and a review of all the products in this market is beyond the scope of this review.

## Morbidity of common cold

As mentioned above, all respiratory viruses are associated with causing a common cold syndrome, but the mild illness can develop into more serious lower respiratory tract infections. Influenza infection has always been associated with significant morbidity and mortality (86) but with the advent of new diagnostic tests for respiratory viruses it has become apparent that all respiratory virus infections have a risk of morbidity and mortality. Respiratory viruses may cause a range of problems such as otitis media, sinusitis, bronchiolitis, pneumonia and exacerbation of respiratory chronic diseases such as asthma, bronchitis and chronic obstructive pulmonary disease (COPD).

Human respiratory syncytial virus (RSV) is a cause of common cold symptoms, but at the extremes of life in infants and the elderly it is a significant cause of morbidity and mortality, causing bronchiolitis in infants and exacerbation of chronic respiratory and cardiac disease in the elderly. Infection with RSV does not give prolonged immunity (87, 88) and infections occur throughout life. RSV is the most common pathogen identified in infants and young children with acute lower respiratory tract infections, and in 2015 it was estimated that there were 33.1 million episodes with 3.2 million hospital admissions world -wide with over 11,800 deaths (89). More recent data on the mortality associated with RSV indicates one death in every 50 attributable to RSV infection in children aged 0–60 months and one death in every 28 for children aged 28 days–6 months (89). RSV causes a substantial burden of disease in those aged over 65 years and in 2015 it was estimated that RSV and other respiratory viruses have caused 1.2 million deaths in the elderly with RSV being the most common cause of death (90).

Rhinoviruses are the most common cause of the common cold and have in the past been considered as more of a nuisance than a serious disease. However, with advances in identifying viruses, there is increasing evidence that rhinoviruses are responsible for considerable morbidity and mortality, especially as regards exacerbation of asthma (91–93). What has become apparent is that respiratory viruses that were believed to be restricted to upper respiratory tract infections due to temperature sensitivity are a common cause of lower respiratory infections (94).

## Vaccines for common cold

The general public consider the common cold as a single disease entity but as stated in the introduction to this review, “because of the large number of respiratory viruses causing colds it is one of the most complex of human diseases”. It is therefore not surprising that development of vaccines for respiratory viruses has focussed on those that are believed to be commonly associated with morbidity and mortality i.e., influenza and RSV. Influenza vaccines were developed in the 1940's as an inactivated virus administered by injection but in later years intranasal live attenuated vaccines have been developed and are used in children (95). Unsuccessful attempts have been made to develop a vaccine for RSV with vaccines paradoxically causing more severe disease in infected infants (96) but recent research has now developed an RSV vaccine for use in the elderly which was approved on May 3rd 2023 by the US Food and Drug Administration for individuals 60 years and older (97).

The SARS-CoV-2 pandemic caused a great surge in interest in vaccine research and the RNA vaccines developed for COVID-19 may provide technology for development of other vaccines for RNA respiratory viruses such as rhinoviruses.

## Discussion

The common cold is not a single disease but is a syndrome of familiar symptoms caused by many different respiratory viruses. The mild symptoms of rhinitis may develop into a lower respiratory tract infection with serious morbidity and mortality in infants, the elderly and those with underlying health conditions such as immune compromised persons. The symptoms of rhinitis associated with respiratory virus infection have some similarity with the symptoms of allergic rhinitis but differ in that histamine is a major mediator in symptoms of allergic rhinitis such as itching and nasal congestion but is not involved in generating the symptoms of common cold (98). The pain and congestion symptoms associated with common cold are mediated by prostaglandins and bradykinin (72).

Because of the familiarity of this common syndrome the complexity and morbidity and mortality of the common cold caused by hundreds of different serotypes of virus is often not fully appreciated. The pandemic caused by SARS-CoV-2 has led to a revolution in research on respiratory viruses and it is interesting to note that with increased exposure and increased immunity to SARS-CoV-2 world-wide, COVID-19 may now evolve to cause a common cold (99).
